# Complete Genome Sequences of Three *Siphoviridae* Bacteriophages Infecting *Salmonella enterica* Serovar Enteritidis

**DOI:** 10.1128/genomeA.00939-16

**Published:** 2016-11-17

**Authors:** Rubina Paradiso, Serena Lombardi, Maria Grazia Iodice, Marita Georgia Riccardi, Massimiliano Orsini, Sergio Bolletti Censi, Giorgio Galiero, Giorgia Borriello

**Affiliations:** a Istituto Zooprofilattico Sperimentale del Mezzogiorno, Portici, Italy; b Cosvitec s.c.ar.l., Naples, Italy; c Istituto Zooprofilattico Sperimentale G. Caporale, Teramo, Italy

## Abstract

Three bacteriophages, 118970_sal1, 118970_sal2, and 64795_sal3, were isolated from water buffalo feces in southern Italy, exhibiting lytic activity against *Salmonella enterica* serovar Enteritidis. These bacteriophages belong to the *Siphoviridae* family and have a 60,113-bp, 123,930-bp, and 48,094-bp double-stranded DNA (dsDNA) genome containing 72, 173, and 80 coding sequences (CDSs), respectively.

## GENOME ANNOUNCEMENT

*Salmonella* is one of the most common causes of foodborne disease worldwide and a frequent etiological agent of diarrhea in swine, poultry, cattle (1), and water buffalo (2). This pathogen resides in the gastrointestinal tract of animals and is excreted in the environment through feces (1). The widespread occurrence of antibiotic resistance urges the need for new approaches against bacterial pathogens. Since bacteriophages can be used as prophylactic agents, we isolated and characterized 3 phages, 118970_sal1, 118970_sal2, and 64795_sal3, from water buffalo feces infected with *Salmonella enterica* serovar Enteritidis.

Phage DNA was purified with the QIAamp DNA minikit, according to the manufacturer’s instructions. DNA sequencing of the bacteriophages 118970_sal1, 118970_sal2, and 64795_sal3 was performed using the Ion Torrent PGM platform, yielding a total of 110,178, 263,893, and 435,128 reads, with average read lengths of 249, 249, and 253 bp and average coverages of 461×, 575×, and 2,428×, respectively. Quality control and trimming were carried out using in-house-implemented Python scripts, assembly was performed by the SPAdes software (3), and finishing was completed using the DraftDoctor software (version 1.0 CRS4; M. Orsini, unpublished data). Genome annotation was manually curated after a preliminary annotation performed by Prokka (4).

The bacteriophages 118970_sal1 and 64795_sal3 exhibited lysogenic characteristics, as shown by plaque morphology. Their genomes consisted of 60,113 bp and 48,094 bp, with G+C contents of 56.5% and 45.6%, respectively. 118970_sal1 contained 72 predicted coding sequences (CDSs), 21 of which matched with identified phage genes (12 genes coding for structural proteins, eight involved in DNA replication, and one with regulatory function). 64795_sal3 contained 80 predicted CDSs, with 25 matching identified phage genes (11 genes involved in DNA replication, nine in phage structure, and five in phage physiology). The remaining CDSs encoded hypothetical proteins. No genes associated with toxin production, *Salmonella* virulence, or antibiotic resistance were identified, while in the 64795_sal3 genome, we identified a gene coding for the gp7 protein, involved in lysogeny (5).

The 118970_sal2 genome consisted of 123,930 bp, with a long terminal repeat of 8,825 nucleotides (nt) and a G+C content of 40.2%. The genome contained 173 predicted CDSs, 30 of which matched identified phage genes, including 14 genes involved in phage structure, 11 genes in DNA replication, and five genes in phage physiology. The remaining 143 CDSs encoded hypothetical proteins. No genes associated with lysogeny, toxin production, *Salmonella* virulence, or antibiotic resistance were identified, therefore suggesting a possible use of this phage as a prophylactic agent for the control of *Salmonella*. Further analysis, however, will be required to assign potential functions to the unidentified genes.

Blast analysis indicated that the three phages belong to the *Siphoviridae* family. The 118970_sal2 genome showed 91% identity (determined with EMBOSS stretcher) to phage Stitch (6) and was therefore classified as a T5-like virus. Phage 64795_sal3 showed 59.3% identity to phage E1 (7), while phage 118970_sal1 showed 92.7% identity to phage χ (8).

### Accession number(s).

The complete genomes of the bacteriophages 118970_sal1, 118970_sal2, and 64795_sal3 have been deposited in GenBank under the accession numbers KU927500, KX017521, and KX017520, respectively.
